# Staurosporine Induces Necroptotic Cell Death under Caspase-Compromised Conditions in U937 Cells

**DOI:** 10.1371/journal.pone.0041945

**Published:** 2012-07-31

**Authors:** Zsuzsanna A. Dunai, Gergely Imre, Gabor Barna, Tamas Korcsmaros, Istvan Petak, Pal I. Bauer, Rudolf Mihalik

**Affiliations:** 1 1st Department of Pathology and Experimental Cancer Research, Semmelweis University, Budapest, Hungary; 2 Institute of Biochemistry II, Medical Faculty of the Goethe University, Frankfurt am Main, Germany; 3 Department of Genetics, Eotvos Lorand University, Budapest, Hungary; 4 KPS Medical Biotechnology and Healthcare Services Ltd, Budapest, Hungary; 5 Department of Medical Chemistry, Molecular Biology and Pathobiochemistry, Semmelweis University, Budapest, Hungary; 6 Department of Medical Biochemistry, Semmelweis University, Budapest, Hungary; 7 Department of Pathogenetics, National Institute of Oncology, Budapest, Hungary; University of Sherbrooke, Canada

## Abstract

For a long time necrosis was thought to be an uncontrolled process but evidences recently have revealed that necrosis can also occur in a regulated manner. Necroptosis, a type of programmed necrosis is defined as a death receptor-initiated process under caspase-compromised conditions. The process requires the kinase activity of receptor-interacting protein kinase 1 and 3 (RIPK1 and RIPK3) and mixed lineage kinase domain-like protein (MLKL), as a substrate of RIPK3. The further downstream events remain elusive. We applied known inhibitors to characterize the contributing enzymes in necroptosis and their effect on cell viability and different cellular functions were detected mainly by flow cytometry. Here we report that staurosporine, the classical inducer of intrinsic apoptotic pathway can induce necroptosis under caspase-compromised conditions in U937 cell line. This process could be hampered at least partially by the RIPK1 inhibitor necrotstin-1 and by the heat shock protein 90 kDa inhibitor geldanamycin. Moreover both the staurosporine-triggered and the classical death ligand-induced necroptotic pathway can be effectively arrested by a lysosomal enzyme inhibitor CA-074-OMe and the recently discovered MLKL inhibitor necrosulfonamide. We also confirmed that the enzymatic role of poly(ADP-ribose)polymerase (PARP) is dispensable in necroptosis but it contributes to membrane disruption in secondary necrosis. In conclusion, we identified a novel way of necroptosis induction that can facilitate our understanding of the molecular mechanisms of necroptosis. Our results shed light on alternative application of staurosporine, as a possible anticancer therapeutic agent. Furthermore, we showed that the CA-074-OMe has a target in the signaling pathway leading to necroptosis. Finally, we could differentiate necroptotic and secondary necrotic processes based on participation of PARP enzyme.

## Introduction

Necrosis is considered as a direct cause or as a simultaneously occurring secondary phenomenon of cell death. Necrosis is important in many human diseases such as neurodegenerative diseases [1], pancreatitis [2], trauma [3], ischemia reperfusion in heart attack [4] or in brain injury [5]. Nevertheless, accumulating evidences have confirmed that necrotic cell death can also be a regulated event and therefore be classified as programmed cell death in line with apoptosis [6]–[11]. A novel, necrotic-like, caspase-independent cell death form has been recently described and termed as necroptosis [12]. Degterev *et al.* demonstrated that stimulation of the extrinsic apoptotic pathway by tumor necrosis factor-alpha (TNFα) or Fas ligand (FasL) under caspase-compromised conditions in certain cell types resulted in a necrotic-like process [12]. This pathway can be hampered by a small molecular weight inhibitor called necrostatin-1 (Nec), which acts by inhibiting the kinase activity of receptor-interacting protein kinase 1 (RIPK1) [13] and by necrosulfonamide (NSA), an inhibitor of mixed lineage kinase domain-like protein (MLKL), substrate of receptor-interacting protein kinase 3 (RIPK3) [14].

The most widely studied pathway leading to necroptosis is triggered by TNFα (see reviews [15], [16]) which is a classical inducer of the extrinsic apoptotic pathway. Tumor necrosis factor receptor 1 (TNF-R1) upon activation by TNFα undergoes rapid conformational changes. Rearrangement of the intracellular part of TNF-R1 provides docking surface for TNFα receptor-associated death domain protein (TRADD) and several different ubiquitin ligases to form the so-called membrane-associated complex I [17]. Polyubiquitination of RIPK1 in complex I contributes to the release of nuclear factor kappa-B (NFκB) and the activation of the pro-survival pathway [8], [18]. If the pro-death signal is stronger or lasts longer than the pro-survival signal, the internalized TNF-R1 and the deubiquitinated RIPK1 form a new cytoplasmic complex. In the cytosolic complex II the activated caspase-8 directs cell to apoptosis and with the cleavage of RIPK1 and RIPK3 prevents the fulfillment of necroptosis. Under caspase-deficient conditions cleavage of RIPK1 and RIPK3 is postponed and, as a consequence, kinase activities of RIPK1 and RIPK3 remain active. Necrosome is formed due to the phosphorylation-driven assembly of RIPK1 and RIPK3 containing complex IIB [17], [19], that subsequently leads to necroptosis [20], [21]. The further downstream events of necroptosis are rather enigmatic (see review [8]). Nowadays, extensive research focuses on the molecular background of necroptosis [22]–[25] and on the identification of necroptosis in physiological [26], [27] or pathological [28], [29] conditions. Recently Tenev *et al.* have shown the receptor- and complex I-independent assembly of Ripoptosome in response to genotoxic stress [25]. Moreover Feoktistova *et al.* confirmed that loss of cIAPs can promote the spontaneous formation of an intracellular platform which is able to activate both apoptosis and necroptosis [23]. For further details see review [24]. Most recently MLKL was identified as the target of RIPK3 [14]. MLKL is phosphorylated by RIPK3 and this step seems to be critical for the fulfillment of necroptosis.

Previously we studied the nature of the switch mechanism between apoptosis and necrosis and investigated the intrinsic apoptotic pathway in staurosporine (STS)-treated U937 cells [30]. STS is a generally accepted inducer of intrinsic apoptotic pathway and it is a wide spectrum inhibitor of protein kinases [31]. STS initiates apoptosis by enhancing mitochondrial permeability transition [32], [33]. In absence of clearing mechanism, apoptotic cell death process is followed by secondary necrosis, disruption of the plasma membrane. Earlier, we [30], [34] and others [35] found that STS could provoke necrosis in caspase-compromised cancer cells. We were interested to examine the role of necroptosis in the same necrotic process, in spite of the fact that necroptosis is generally defined as a result of a death receptor-triggered cell death pathway [12].

In this report we aimed to characterize the STS-induced necrosis in U937 cell line. We were curious about the role of RIPK1, poly(ADP-ribose)polymerase (PARP) and the effect of lysosomal enzyme inhibitor CA-074-OMe in STS-triggered necrosis to differentiate secondary necrosis and necroptosis [34]. We also focused on the tumor necrosis factor-related apoptosis-inducing ligand (TRAIL)-induced necroptotic type of cell death that might has high clinical relevance because of the known ability of TRAIL cytokine to induce apoptosis in a variety of human cancer cell lines while leaving most of normal cells unaffected [36]. We concluded that both TRAIL and STS can induce at least partially necroptosis which can be completely hampered by CA-074-OMe but not by the PARP inhibitor PJ-34.

## Results

### TRAIL Induces Necroptosis in U937 Cell Line in the Presence of a Caspase Inhibitor

To build a reliable model system, first, we tested the classical death ligand-induced necroptosis with TRAIL cytokine. TRAIL is known to induce apoptosis in a wide variety of tumor cells [37], [38] and necroptosis in Jurkat cells [39]. We selected U937 monocytic cell line which was shown to undergo necroptosis induced by TNFα in the presence of a caspase inhibitor [12].

**Figure 1:**
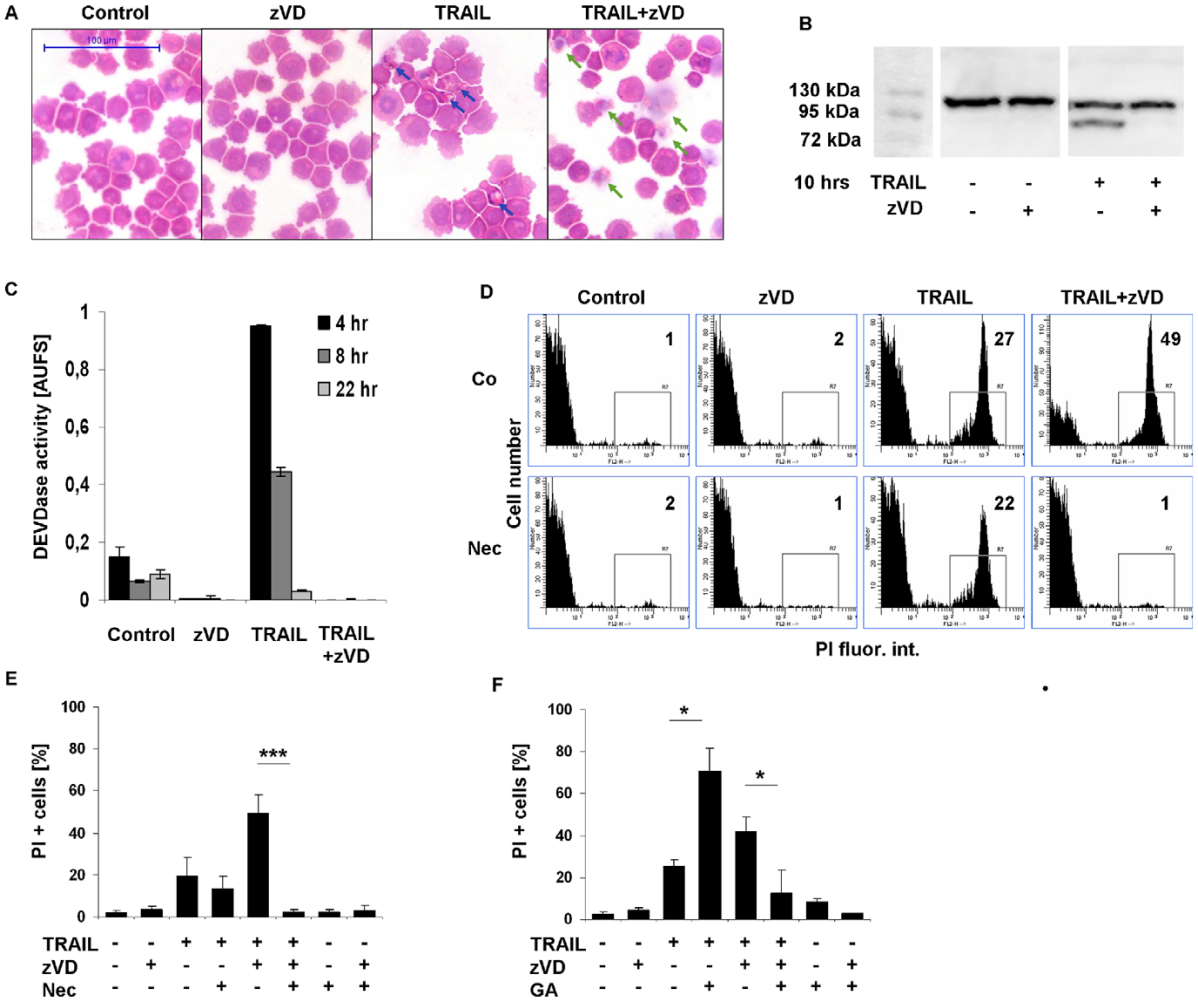
TRAIL induces necroptosis in the presence of caspase inhibitor. TRAIL-induced cell death in U937 cells. Cells were treated as indicated for 20 hrs. (A) Cytospins were stained with hematoxylin-eosin. Blue arrows show the apoptotic, green ones the necrotic cells (400x) (representative of n = 2). Scale bar on the first subfigure applies to all the figures in the panel. (B) Western blot analysis of fragmented PARP-1 protein. Full length PARP-1 is 116 kDa, cleaved PARP-1 fragment is 89 kDa (representative of n = 2). (C) TRAIL-induced caspase activity in U937 cells. U937 cells were exposed to 50 ng/mL hr-TRAIL (114–281 aa) in the presence or absence of zVD (5 µM) for the indicated period of time. The ordinate shows the slope of the measured DEVDase activity curves of a representative experiment carried out in triplicates. (D) FACS analysis of treated cells. Plasma membrane integrity was analyzed after PI staining of cells. Inserted values on histograms show the percentage of the marked population (representative of n = 7). (E-F) Nec and GA protected U937 cells from TRAIL+zVD-induced necroptosis. U937 cell were exposed to TRAIL (50 ng/mL) in the presence or absence of zVD (5 µM) and (E) Nec (10 µM) or (F) GA (1 µM) for 20 hrs. PI stained cells were analyzed for membrane permeability. Percentages of PI positive cells were determined (n = 7 for Nec and n = 4 for GA). Values are mean±SD. *, P<0.05, **, P<0.01 and ***, P<0.001 calculated by Student’s t-probe.

We found U937 cells sensitive to TRAIL cytokine treatment. Light microscopic studies of hematoxylin-eosin stained samples confirmed that TRAIL treatment generated apoptotic nuclear DNA condensation (Fig. 1A) together with nucleosomal DNA fragmentation revealed by flow cytometric sub-G1 assay method (Fig. S1). These changes resulted in apoptotic PARP-1 fragmentation (Fig. 1B) due to caspase-3 activation (Fig. 1C). Finally, signs of secondary necrosis occured when cells turn permeable for propidium iodide (PI) (Fig. 1D, 1E).

When cells were pre-treated with the pan-caspase inhibitor z-VD.fmk (zVD) one hour before TRAIL treatment, the executioner caspase activation was prevented (Fig. 1C), therefore PARP-1 processing was inhibited (Fig. 1B). Apoptotic type oligonucleosomal DNA fragmentation diminished, however, the high molecular weight fragmentation of DNA appeared resembling necrosis [40], [41] (Fig. S1) and DNA condensation was evaded (Fig. 1A). Instead of apoptotic morphology, dying cells were swelling and showing a “ghost-like” morphology resembling necrosis (Fig. 1A). Plasma membrane rupture detected and quantified by PI uptake revealed that the number of PI positive cells in caspase inhibitor-sensitized U937 cells upon TRAIL treatment were increasing compared to cells treated with TRAIL alone (Fig. 1D, 1E). In the presence of RIPK1 inhibitor Nec (10 µM), PI uptake was reduced only marginally in caspase-competent (secondary necrotic) cells, while it was completely reduced in caspase-compromised cells after TRAIL treatment (Fig. 1D, 1E).

To further confirm the role of RIPK1 activity in TRAIL-induced necroptotic or secondary necrotic cell death processes we studied the effect of geldanamycin (GA, 1 µM). GA, an inhibitor of the heat shock protein 90 kDa (HSP90) [42], was known to downregulate the protein level of RIPK1 [43] and therefore was expected to halt necroptosis. Indeed, GA pre-treatment (4 hrs) significantly reduced the extent of TRAIL-induced necroptosis detected by PI uptake (Fig. 1F). At the same time GA enhanced the extent of TRAIL-induced apoptosis (Fig. S1), possibly via downregulating the NFκB signaling pathway [44]. Furthermore GA caused cell cycle inhibition in G2 phase (Fig. S1). Secondary necrosis (Fig. 1F) was also increased in line with the elevation of DNA fragmentation (Fig. S1).

These results indicate that in the presence of caspase inhibitor, TRAIL-exposed U937 cells undergo necroptosis instead of apoptosis, moreover TRAIL-triggered necroptosis can be suspended both by Nec and GA.

### STS Induces Primary Necrosis in the Presence of a Caspase Inhibitor

**Figure 2:**
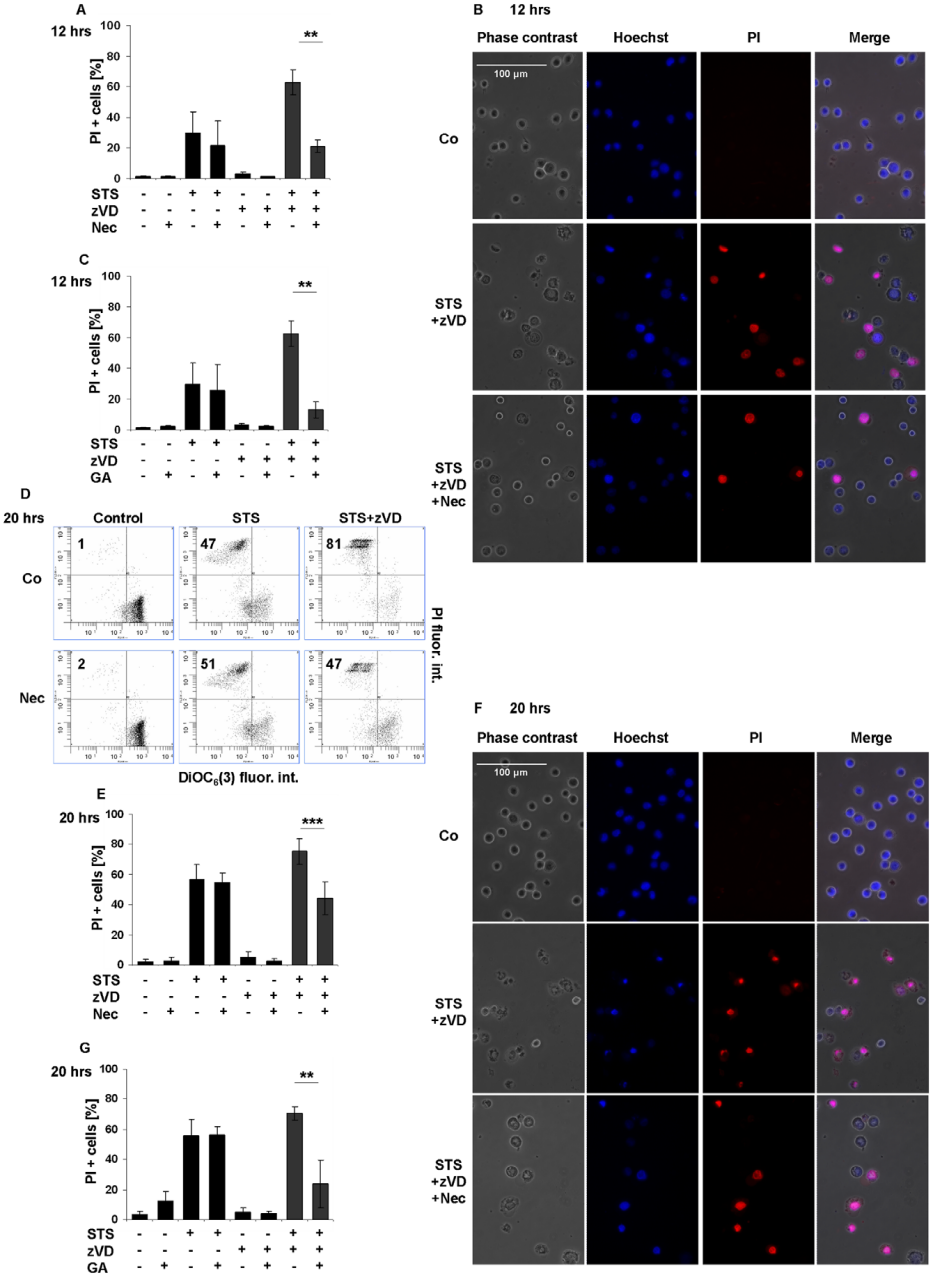
STS induces primary necrosis in the presence of caspase inhibitor. (A-B) Nec (10 µM) and (C) GA (1 µM) significantly inhibited the STS-triggered necroptosis. Cells were exposed to STS (1 µM) in the presence or absence of zVD (5 µM) for 12 hrs. Percentage of PI positive cells was determined by flow cytometry (A, C). (n = 4) and by Hoechst/PI double staining technique (B) (representative of n = 2), (400x). Scale bar on the first subfigure applies to all the figures in the panel. (D) Nec (10 µM) arrested the STS-induced (1 µM) necroptosis in the presence of zVD (5 µM) after 20 hrs incubation. The mitochondrial transmembrane potential and plasma membrane integrity is shown in representative dot plots of DiOC_6_(3) and PI stained, unfixed cells. The values indicate the percentage of cells in the marked regions (n = 13). (E-G) Nec (10 µM) (E, F) and GA (1 µM) (G) partially inhibited the STS-triggered necroptosis. Cells were exposed to STS (1 µM) in the presence or absence of zVD (5 µM) for 20 hrs. Percentage of PI positive cells was determined by flow cytometric analysis (E, G) (n = 13 for Nec and n = 4 for GA), and by Hoechst/PI double staining technique (F) (n = 2). Scale bar on the first subfigure applies to all the figures in the panel. Values are mean±SD. *, P<0.05, **, P<0.01 and ***, P<0.001 calculated by Student’s t-probe.

To study the STS-evoked necrosis we tested the effect of the RIPK1 inhibitor under caspase-compromised conditions. Nec concentration dependently abrogated the necrosis confirmed by the reduced ratio of PI positive cells (Fig. S2A). At the applied concentration Nec (10 µM) and GA (1 µM) arrested the necrosis after 12 hrs of incubation time confirmed by flow cytometry and by fluorescent microscopic studies using Hoechst dye and PI double staining technique (Fig. 2A, 2B, 2C). After prolonged (20 hrs) treatment the STS and zVD-triggered plasma membrane rupture was diminished only partially by Nec (Fig. 2D, 2E, 2F) or GA (Fig. 2G). Furthermore, Nec unaffected the caspase-mediated DNA fragmentation and condensation either after shorter (8 hrs) (Fig. S2B, S2C) or after longer incubation time (20 hrs) (Fig. S2D, S2E). GA affected only moderately the DNA fragmentation (Fig. S2F) delaying the appearance of the shorter nucleosomal fragments (Fig. S2H). The STS-induced secondary necrosis, the final stage of cell death, was unaffected by either Nec (Fig. 2E) or GA (Fig. 2G).

Concerning the apoptotic parameters, zVD prevented the executioner caspase activation (Fig. S2G). It is worth to mention that the proportion of sub-G1 cells was reduced both by Nec (Fig. S2E) and by GA (Fig. S2F) after prolonged STS treatment in the presence of zVD. However this observed reduction correlated well with the reduced ratio of the number of PI positive cells (Fig. 2E, 2G). This correlation denotes that the number of sub-G1 DNA containing cells observed when STS and the caspase inhibitor were applied, is unequal with the classical fragmented DNA containing population obtained after STS treatment alone. STS and zVD-exposed samples showed high molecular weight DNA fragments confirmed by agarose gel electrophoresis, accompanied with low molecular weight DNA fragments that are primarily smear-like, opposite to apoptotic DNA fragmentation and typical to necrosis [40], [41]. STS treatment resulted in nucleosomal DNA ladder fragmentation trimmed by postmortem DNase activity. (Fig. S2H).

These results indicate that STS-induced apoptosis in U937 cells ensued by secondary necrosis, while under caspase-compromised conditions STS induced primary necrosis that is partially inhibitable by Nec and GA, two drugs affecting RIPK1 activity.

### STS and TRAIL Induce RIPK1 and MLKL-dependent Necroptosis

**Figure 3:**
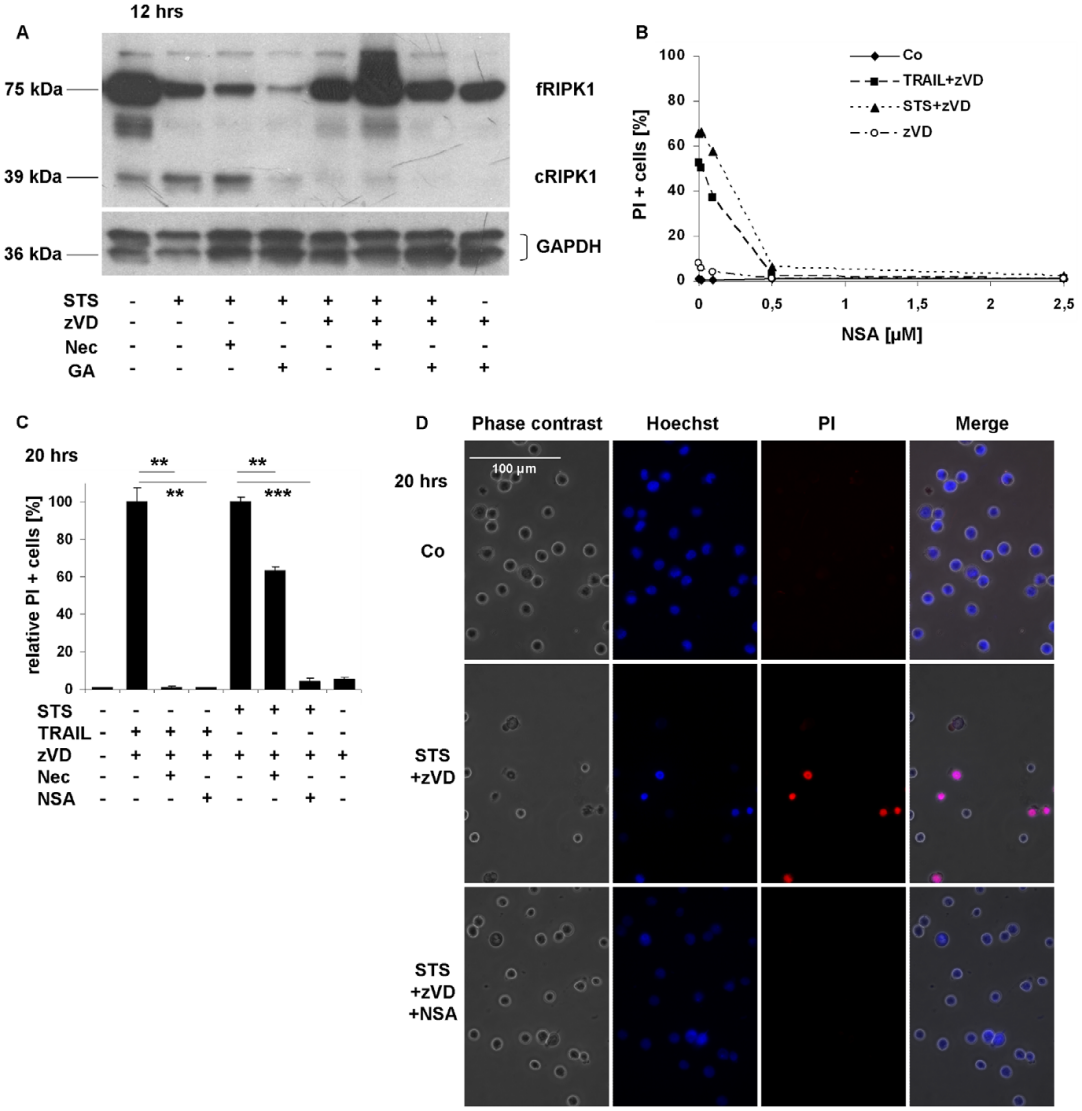
STS induces RIPK1 and MLKL-dependent necroptosis. (A) zVD hampered RIPK1 fragmentation triggered by STS for 12 hrs. Western blot analysis was performed for the detection of RIPK1 protein level and presence of cleaved fragment due to caspase activity (representative of n = 2). (B) NSA reduced TRAIL and STS-induced necroptosis in a concentration-dependent manner after 20 hrs incubation time – representative experiment. U937 ells were exposed to STS (1 µM) and TRAIL (50 ng/mL) and varying concentrations of NSA (0–2.5 µM) in the presence of zVD (5 µM) for 20 hrs. Percentage of PI positive cells was determined. (C-D) NSA (0.5 µM) significantly hampered both the TRAIL and STS-triggered necroptosis. (C) Relative percentage of PI positive cells was determined by FACS analysis (n = 3). Values are mean±SD. *, P<0.05, **, P<0.01 and ***, P<0.001 calculated by Student’s t-probe. (D) Morphological signs of apoptosis and necrosis are shown in representative fluorescent microscopic images (400x) of Hoechst/PI double stained U937 cells (n = 2). Scale bar on the first subfigure applies to all the figures in the panel.

To confirm the role of RIPK1 in the STS-induced necroptosis, we tested the proteolytic degradation of RIPK1 in U937 cell line by Western blot analysis. STS-triggered caspase activation led to RIPK1 processing (Fig. 3A). Full length RIPK1 (∼75 kDa) was proteolytically cleaved by caspase-8 during STS-induced apoptosis [45]. The cleavage at the carboxyl group of aspartic acid residue (at amino acid position of 324) resulted in two fragments, a shorter N-terminal part which contains the kinase domain (∼37 kDa) and a longer C-terminal fragment with the RIP homotypic interaction motif (RHIM) and death domain (∼39 kDa) [46]. Processing of RIPK1 was highly inhibited in the presence of the caspase inhibitor zVD (Fig. 3A).

To further study the molecular components involved in STS-triggered necroptotic pathway under caspase-compromised conditions we investigated the role of MLKL. Cells were treated with various concentrations of NSA, an inhibitor of MLKL [14]. NSA reduced the ratio of PI positive cells in a concentration-dependent fashion independently of the way of cell death trigger (either TRAIL or STS) under caspase-compromised condition (Fig. 3B). Complete inhibition was observed at NSA concentration of 0.5 µM, the same concentration as reported by Sun *et al.* [14]. NSA at the applied concentration arrested both the TRAIL and STS-triggered necrosis, confirmed by PI uptake measurements (Fig. 3C) and also by Hoechst/PI double staining method (Fig. 3D). Meanwhile NSA failed to reverse the STS-evoked DNA fragmentation (Fig. S3A, S3B) or prevent secondary necrosis (Fig. 3SB).

These results confirmed the crucial role of RIPK1 and MLKL in STS and TRAIL-induced necrosis under caspase-compromised conditions. Therefore we can consider the observed necrosis in U937 cells as necroptosis.

### CA Inhibits both the TRAIL and STS-induced Necroptosis in the Presence of a Caspase Inhibitor

Previously we found that CA-074-OMe (CA), an inhibitor of cysteine cathepsins, rescued the caspase-independent necrotic form of cell death of promyelocytic leukemia cells treated by STS [34]. In this study we tested whether CA (10 µM) treatment might promote the survival of U937 cells dying under necroptotic conditions.

**Figure 4:**
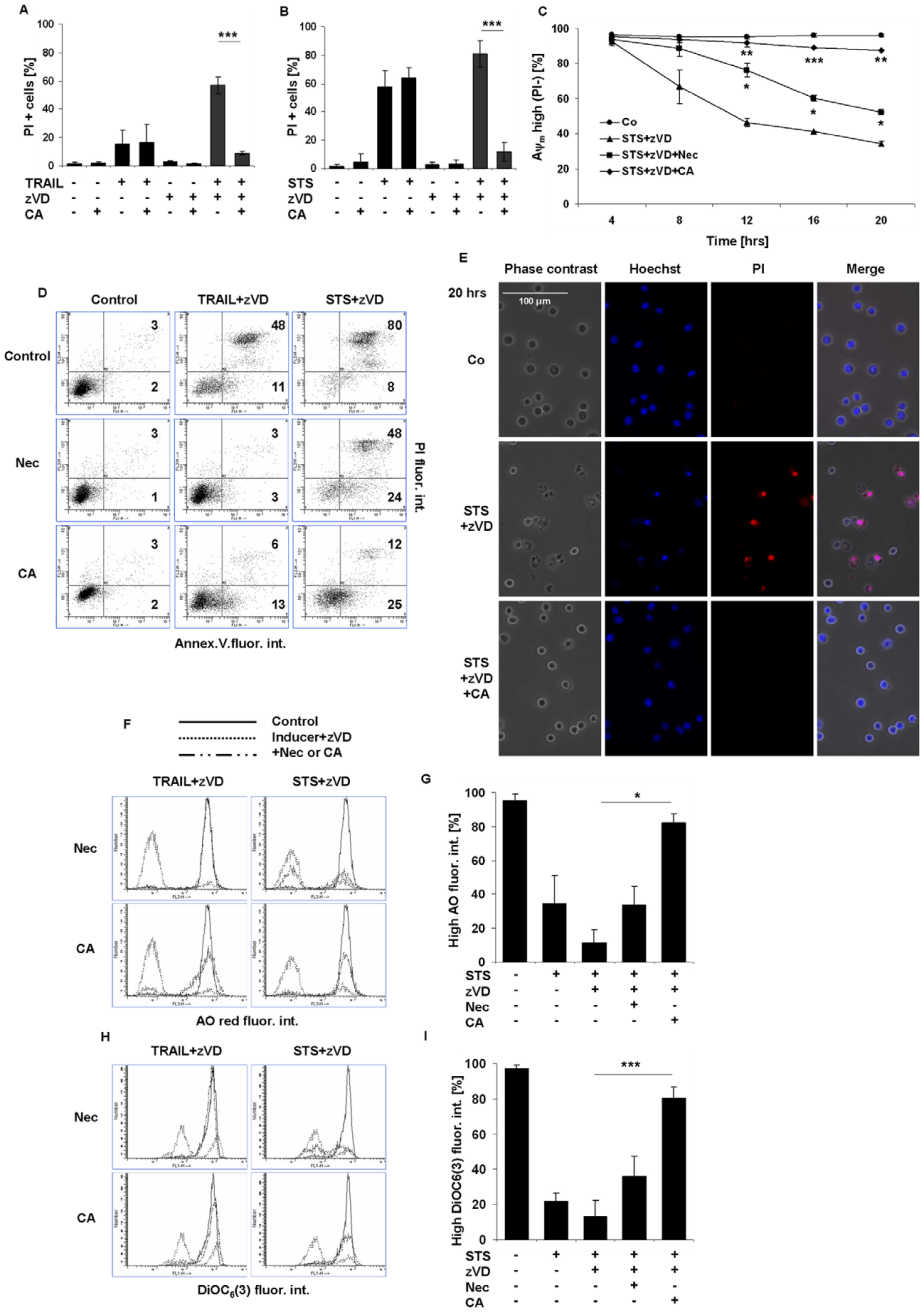
CA inhibits either the TRAIL or STS-induced necroptosis in presence of caspase inhibitor. U937 cells were treated either with STS (1 µM) or with TRAIL (50 ng/mL) in the presence or absence of zVD (5 µM) for 20 hrs. Nec (10 µM) or CA (10 µM) were added 1 hr before cell death was induced. (A-B) CA (10 µM) considerably inhibited the (A) TRAIL (n = 4) or (B) STS-triggered necroptosis (n = 3). Percentage of PI positive cells was determined. (C) Time course analysis of cells with depolarized mitochondria is shown after DiOC_6_(3) staining of, unfixed cells for STS treatment combined with the indicated inhibitors (n = 2). Values are mean±SD. *, P<0.05, **, P<0.01 and ***, P<0.001 calculated by Student’s t-probe. (D) PS distribution in the plasma membrane is shown in representative dot plots of Annexin V-FITC and PI stained, unfixed cells analyzed by flow cytometry. The values indicate the percentage of cells in the marked regions (n = 2). (E) Morphological signs of apoptosis and necrosis are shown in representative fluorescent microscopic images (400x) of Hoechst/PI double stained U937 cells (n = 2). Scale bar on the first subfigure applies to all the figures in the panel. (F) Distribution of cells with various volumes of acidic compartments (endo-lysosomes) is shown in representative overlay histograms of AO stained cells analyzed by flow cytometry. (G) Column diagram of percentage of cells with high AO red fluorescence intensity (n = 2). (H) Distribution of cells with various mitochondrial transmembrane potential is shown in representative overlay histograms of DiOC_6_(3) stained cells analyzed by flow cytometry. (I) Column diagram of percentage of cells with high DiOC_6_(3) fluorescence intensity (n = 3). Values are mean±SD. *, P<0.05, **, P<0.01 and ***, P<0.001 calculated by Student’s t-probe.

Interestingly CA almost completely hampered the TRAIL-induced necroptotic cell death measured by plasma membrane rupture (Fig. 4A). Moreover, CA was comprehensive inhibitor of STS-triggered necroptosis too (Fig. 4B). In case of both inducers, CA abolished the ratio of PI positive cells in a concentration-dependent manner (Fig. S4A, S4B). IC_50_ amount was approximately at 5 µM in both cases (Fig. S4A, S4B). To further characterize the effect of CA on the necroptotic pathway and to compare to the inhibitory activities of Nec and GA, its action on different cellular compartments were investigated. Time course detection of mitochondrial membrane depolarization was carried out by flow cytometric analysis of DiOC_6_(3) stained STS+zVD-treated U937 cells. Our results confirmed that a continuous, time-dependent reduction of mitochondrial membrane integrity happens in the cell population (Fig. 4C). While Nec prevented partially the cells to loose their mitochondrial membrane potential for STS-treatment, CA provided nearly complete protection under caspase activity arrested conditions (Fig. 4C). Furthermore Nec completely inhibited the appearance of AnnexinV and PI double positive cell population after TRAIL and zVD co-treatment (Fig. 4D). In case of STS treatment, the depletion was just partial (Fig. 4D) in concordance with the earlier mentioned PI positivity results (Fig 2D, 2E). Contrarily, the effect of CA treatment on the reduction of phosphatidylserine (PS) translocation and plasma membrane rupture was complete (Fig. 4D). Similarly, the complete arrest of CA on plasma membrane rupture was detected by PI uptake (Fig. 4A, 4B) and by Hoechst/PI double staining technique for STS (Fig. 4E) or TRAIL (data not shown) treated U937 cells in the presence of zVD. Moreover the loss of lysosomal acidity induced either by STS (Fig. 4F, 4G) or TRAIL (Fig. S4C) treatment under necroptosis inducing conditions was remarkably withheld by CA pretreatment. Additionally CA could significantly prevent the loss of mitochondrial membrane depolarization in both cases of STS (Fig. 4C, 4H, 4I) and TRAIL (Fig. 4H) treatment in the presence of the caspase inhibitor zVD.

Compared to the aforementioned effects of CA, Nec was only partially effective inhibitor of all the measured cellular functions in STS-triggered necroptosis (Fig. 4C, 4D, 4F, 4G, 4H, 4I). On the other hand Nec completely diminished the TRAIL-induced necroptosis (Fig. 4D, 4F, 4H, Fig. S4C, S4D). The observed apoptotic parameters: the invariable percentage of the sub-G1 cell population (Fig. S4E, S4F, S4G) and the presence of ladder type DNA degradation observed in agarose gels (Fig. S4H) proved that CA had no effect on caspase-dependent apoptosis or on the ensuing secondary necrosis (Fig. 4A, 4B) in TRAIL or STS-exposed cells. Moreover, the absence of proteolytic degradation of RIPK1 also confirmed the avoidance of apoptosis (Fig. S4I).

From these data we surmised that CA, similarly to NSA is also an effective inhibitor of the necroptotic pathway induced by STS in the presence of caspase inhibitor. While Nec and GA withheld only the early phase of STS-induced necroptosis in U937 cells.

### PJ-34 does not Arrest Either the TRAIL or STS-induced Necroptosis in the Presence of a Caspase Inhibitor

Participation of PARP-1 enzyme is well documented at several levels of cell death processes [6]. Applying PARP inhibitor reduces the rate of PARP activation and slows down the exhaustion of nicotinamide adenine dinucleotide (NAD) and ATP pools, therefore generally shifts the cell death process towards apoptosis and prevents necrosis [15].

**Figure 5:**
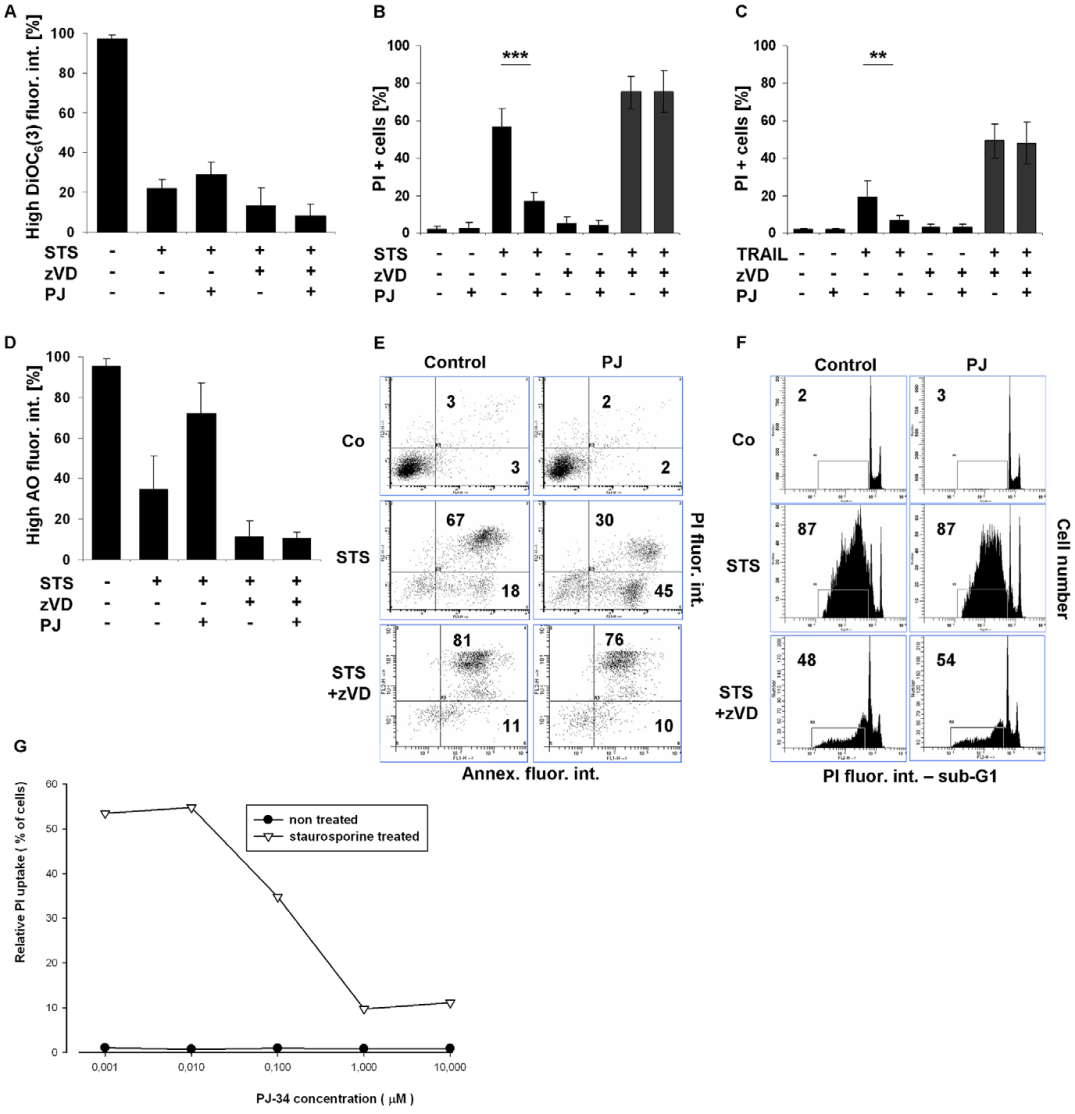
PJ3-4 does not arrest either the TRAIL or STS-induced necroptosis in presence of caspase inhibitor. U937 cells were treated either with STS (1 µM) or with TRAIL (50 ng/mL) in the presence or absence of zVD (5 µM) for 20 hrs. PJ-34 (1 µM) was added 1 hr before cell death was induced. (A) Column diagram of percentage of cells with high DiOC_6_(3) fluorescence intensity (n = 3). (B-C) PJ-34 (1 µM) considerably inhibited the (B) STS (n = 8) or (C) TRAIL-triggered (n = 8) secondary necrosis but not the necroptosis. Percentage of PI positive cells was determined. Values are mean±SD. *, P<0.05, **, P<0.01 and ***, P<0.001 calculated by Student’s t-probe. (D) Column diagram of percentage of cells with high AO fluorescence intensity (n = 2). (E) Dot plot distribution of cells stained with Annexin V-FITC and PI analyzed by flow cytometry. The values indicate the percentage of cells in the marked regions (representative of n = 2). (F) Representative histograms of distribution of PI stained, ethanol-fixed (sub-G1) cells were analyzed by flow cytometry (n = 8). The numbers indicate the percentage of cells in the marked regions. (G) PJ-34 concentration dependently reduced the proportion of PI positive cells for STS treatment for 20 hrs – representative experiment.

In our experimental system we tested the effect of PARP-1 enzyme inhibitor PJ-34 on the necroptotic cell death pathway. Major differences in the necroptotic process were not observed in the presence of the inhibitor. In STS or in TRAIL-treated and caspase-inhibited U937 cells PJ-34 did not influence the changes observed in mitochondrial transmembrane potentials (Fig. 5A, Fig. S5A), in PI permeability of cells (Fig. 5B, 5C), in the acidity of lysosomes (Fig. 5D, Fig. S5B) or in the PS distribution in the plasma membrane (Fig. 5E, data not shown). Furthermore, neither the caspase-dependent nor the caspase-independent STS or TRAIL-induced apoptotic DNA fragmentation was abolished by the applied PARP-1 inhibitor (Fig. 5F, Fig. S5C).

At the same time the secondary necrotic cell death process was postponed by PJ-34 in a concentration-dependent manner. This effect was confirmed by the observed intact plasma membrane integrity both under STS (Fig. 5B, 5G) and TRAIL-induced (Fig. 5C) conditions where the enzyme activity of PARP-1 was increased in the absence of the inhibitor (unpublished results). In addition, the loss of lysosomal acidity was notably reduced by PJ-34 during prolonged treatment of U937 cells with STS (Fig. 5D) and this effect, although with much less extent was observed after TRAIL treatment as well (Fig. S5B).

Contrary to the necrotic process, in cases of STS or TRAIL-induced apoptosis PJ-34 showed only marginal effect on the cell death process. The hallmarks of apoptosis including PS distribution (Fig. 5E, Fig. data not shown), loss of mitochondrial transmembrane potential (Fig. 5A, Fig. S5A) and the DNA fragmentation (Fig. 5F, Fig. S5C) were not influenced by PJ-34. Note, that in Figure 5E most STS-treated cells are Annexin and PI positive while in the presence of PJ-31 more than half of the cells were not stained with PI indicated that PJ-34 prevented postapoptotic membrane disruption.

These results indicate that PJ-34 could only delay the secondary necrosis, but was unable to prevent the caspase-dependent apoptosis. Our results show that the enzymatic activity of PARP-1 directly or indirectly influences the process of secondary necrosis but is dispensable in necroptosis at least in TRAIL and STS-treated U937 cells.

## Discussion

In this report we analyzed different aspects of cell death modalities in TRAIL and STS-treated monocytic U937 cells by using known inhibitors of the apoptotic, necrotic and necroptotic processes. We have found that under caspase-compromised conditions necroptosis can be triggered both by TRAIL and STS, which compounds are classical inducers of the extrinsic and intrinsic apoptotic pathways respectively. Necroptosis could be arrested by the MLKL inhibitor NSA, by the RIPK1 inhibitor Nec, by the HSP90 inhibitor GA and by the cathepsin B inhibitor CA. On the other hand PJ-34, a PARP-1 inhibitor did not affect the necroptotic pathway, but effectively arrested the progress of secondary necrosis ensued the caspase-mediated apoptosis, at least in U937 cell line.

First, we built up a model system to study the classical death ligand-triggered necroptosis. We selected TRAIL cytokine as inducer of cell death which compound is a promising anticancer agent. Although, it was investigated as a potent and selective apoptosis inducing agent in malignant cells [36], its effect on the necroptotic process was not known in detail. In U937 human monocytic cell line TRAIL induced apoptotic cell death with a moderate intensity, characterized by caspase activation, PARP-1 cleavage and ladder type DNA degradation. After extended periods of TRAIL treatment, apoptosis was followed by a secondary necrotic process. Applying zVD pan-caspase inhibitor together with TRAIL, turned the apoptotic process into a necroptotic cell death form that was arrested by Nec, and none of the signs of apoptosis or that of secondary necrosis were observed even after 20 hours of treatment. These results are in concordance with recent publications [13], [39]. After confirming that U937 cells can show apoptotic, necroptotic or secondary necrotic phenotypes upon TRAIL treatment, we started to study the effect of inhibitors influencing certain segments of cell death pathways.

We tested the effect of GA, an inhibitor of the ATP-ase activity of the chaperone protein HSP90. U937 cells were pre-treated with GA (4 hrs) or Nec (1 hr) both in the absence and presence of zVD before TRAIL administration, and the changes of biochemical events were recorded. Nec or GA prevented the rupture of plasma membrane, due to the inhibition of RIPK1 kinase activity by Nec and partial downregulation of RIPK1 protein level by GA in line with the literature [39], [47]. Nec did not affect the extent of TRAIL-induced apoptosis or secondary necrosis in the presence of caspase activation. However, GA administration increased the ratio of apoptotic U937 cells possibly by decreasing the RIPK1-dependent NFκB activation [43]. Previously RIPK1 was described to serve as an adapter surface having critical role in the activation of apoptotic and in NFκB pathways, where its kinase activity is dispensable [48]–[50]. Contrarily, kinase activities of RIPK1 and RIPK3 are crucial for necroptosis [13], [20], [21].

Previously we published that STS induced necrosis in U937 cells where caspase activities were halted by a broad spectrum caspase inhibitor [30]. Detection of early plasma membrane rupture, morphological characteristics and the absence of caspase activities were considered as important signs of necrotic cell death. In the current study we asked if the STS-induced cell death measured in the absence of caspase activities can be considered as necroptosis.

We detected that the STS-induced, caspase activity-independent necrotic-like cell death was also withheld by Nec and GA pre-treatments. This inhibitory action was almost complete after 12 hours and was partial but still significant if the incubation time was longer (20 hrs). Further supporting information is available in Text S1. The reason of the incomplete inhibition of Nec or GA observed after extended periods of treatments with STS and zVD might be connected to alternatively activated cell death pathways (e.g. autophagy). Further supporting information is available in Text S2.

RIPK1 was processed in the STS-induced apoptosis that was dominantly inhibited by the caspase inhibitor zVD. The size of the fragment is in accordance with the caspase-8-mediated cleavage of RIPK1 [51]. As the STS-triggered necroptosis could proceed only in the presence of caspase inhibition, the absence of RIPK1 degradation also supports its role in the STS-evoked necroptosis. Additional supporting information is available in Text S3. To further investigate the possible members of the STS-provoked necroptosis we tested the involvement of MLKL in the process. Our results show that both the TRAIL and the STS-induced necroptosis were prevented by NSA. This observation suggests that not only RIPK1 but MLKL are also involved in the STS-triggered necroptotic pathway. As Nec provides only a partial inhibition while NSA results in full protection we propose the hypothesis that STS acts through two parallel pathways which are equally dependent on MLKL.

How STS induces necroptosis is rather enigmatic. Death receptor-independent assembly of Ripoptosome is a recently proposed model [24]. This process is controlled by endogenous inhibitors of apoptosis proteins (cIAPs) and the X-linked inhibitor of apoptosis protein (XIAP) as they can directly ubiquitinate the components of Ripoptosome [25]. Tenev *et al.* found that during etoposide-induced genotoxic stress the levels of cIAPs and XIAP decreased causing the spontaneous formation of the Ripoptososme [25]. Conceivably during STS-induced necroptosis the cIAP and XIAP level also decrease and therefore the formation of Ripoptosome can occur. This notion is supported by former publications revealing that STS decreases the level of XIAP in leukemia cells [52] and in MCF-7 cells [53].

Previously, cathepsin B, a ubiquitous lysosomal cysteine protease was shown to be a component of TNFα-induced cell death pathway [54]. In the absence of caspase activity cathepsins were shown to process cellular proteins leading either to apoptosis [55] or necrosis [56]. We previously discussed that CA-074-OMe, the methylated variant of a highly specific inhibitor of cathepsin B [57] abrogated the necrotic cell in caspase-inhibited leukemia cells, independently from its inhibitory effect on cathepsin B [34]. In this study we demonstrated that CA stabilized the acidic pH of the lysosomal compartment, prevented the membrane potential loss of mitochondria and finally the rupture of plasma membrane. This indicates that the target of CA is functionally located upstream of the lysosomal breakdown and the mitochondrial depolarization [34]. Van den Berghe and his colleagues recently found that CA significantly blocked TNFα-induced necroptosis [58]. Our results with TRAIL and STS demonstrated that CA completely abrogated necroptosis at 10 µM concentration in the caspase-inhibited U937 cells. However, this CA concentration was higher with orders compared to concentration required for inhibition of cathepsin B protease activity [34], [59]. Thus the effective target of CA in necroptosis remains to be determined.

Participation of PARP and the effect of PARP inhibitor on the prevention or during the induction of cell death are very much cell type dependent. Inhibition of PARP activity might prevent cell death via preventing the exhaustion of a cell’s ATP and NAD pools or by inhibiting the transfer of AIF from mitochondria to cell nuclei. In U937 cells, PARP inhibitor did not influence either the apoptotic or the necroptotic cell death pathways induced either by TRAIL or STS, but were able to postpone the secondary necrotic process. Further supporting information is available in Text S4.

**Figure 6:**
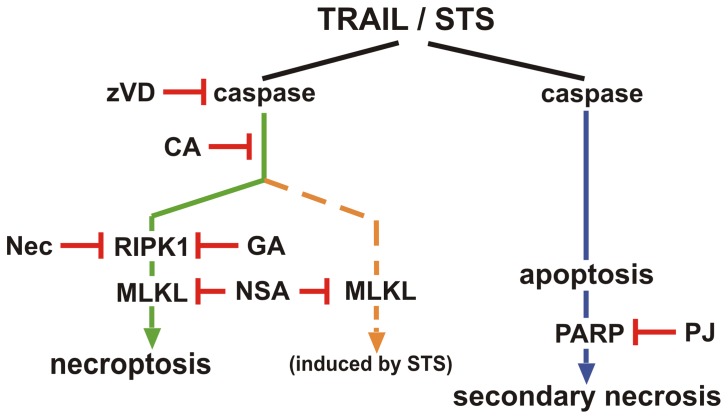
Schematic diagram about the action of inhibitors on TRAIL and STS- induced cell death pathways. Both TRAIL and STS triggered necroptosis in caspase depleted U937 cells upon prolonged incubation time. RIPK1 inhibitors Nec and GA could completely inhibit the TRAIL-evoked necroptosis, but had partial inhibitory potential to the STS-provoked process. Contrarily CA and NSA abolished both the TRAIL and STS-induced necroptosis. In absence of caspase inhibitor TRAIL and STS induced apoptosis which is followed by secondary necrosis. PJ-34 delayed the necrotic plasma membrane disruption during secondary necrosis, but failed to inhibit necroptosis.

In this publication we proved that TRAIL, a death receptor ligand cytokine, which generally induces apoptotic cell death through the extrinsic pathway, under caspase compromised conditions trigger necroptosis in U937 cells. We have also shown that STS, a kinase inhibitor compound which generally induces apoptosis through the intrinsic pathway can also induce necroptosis under caspase-deprived conditions. Our results might have clinical relevance in the fight against tumors. Treatment with proper inductors can activate the necroptotic cell death form in apoptosis resistant tumor cells. Moreover applying PARP inhibitors to delay secondary necrosis might control the tumor lysis syndrome, where the intracellular content spilling out from the abruptly dying large number of cells poisoning the whole body. Furthermore we have shown that CA inhibits the necroptotic cell death. This result point out that the currently unknown target of CA can pharmacologically be important in necroptotic cell death. The flow chart shown in Figure 6 summarizes the hypothetic places of actions, where the drugs were used in the current study to inhibit the STS or TRAIL-induced cell death pathways in U937 cells.

We hope that the presented results will contribute to the better understanding of the molecular background of necroptosis and propagate research on CA to find novel drug candidates against necroptotic cell death.

## Materials and Methods

### Materials

Staurosporine, PARP inhibitor (PJ-34), geldanamycin (GA), RNase A, propidium iodide (PI), DiOC_6_(3), acridine orange (AO), Hoechst 33258 and cell culture products were purchased from Sigma-Aldrich (St. Louis, MO, USA). hr-TRAIL (114-281 aa) was prepared as described earlier [60]. z-Val-DL-Asp-fluoromethylketone (zVD) and CA-074-OMe (CA) were purchased from Bachem (Bubendorf, Switzerland). Necrostatin-1 (Nec) was obtained from Calbiochem (Darmstadt, Germany). Antibody against PARP-1 was ordered from Cell Signaling Technology, (Danvers, MA), (#9542), against RIPK1 from Becton Dickinson (Budapest, Hungary), (610458) and against GAPDH from Santa Cruz (Heidelberg, Germany, (D-6) sc-166545). Necrosulfonamide (NSA) was a kindly gift of Professor Xiaodong Wang (Beijing, China).

### Cell Culture

U937 promonocytic cell line was purchased from ATCC. Cells were cultured in RPMI 1640 supplemented with 10% heat inactivated fetal bovine serum, 2 mM _L_-glutamine and 100 µg/mL penicillin and 100 units/ml streptomycin at 37°C in a 5% CO_2_ containing, humidified incubator. Cells were regularly tested for the presence of certain human CD antigens: CD45 (+); CD33 (+); CD13 (+); CD34 (−); CD14 (−); CD4 (−). Antibodies directly labeled with fluorochrome were purchased from Becton-Dickinson (Franklin Lakes, NJ USA). For treatments, cells were distributed into 48-well suspension plate (Greiner), 0.5 mL into each well at a cell density of 3×10^5^ cells/mL or 5×10^5^ cells/mL, 60 minutes before drug treatment. Then zVD (10 µM) was added with or without together other drugs as described above started 1 hr (Nec, CA, NSA, PJ) or 4 hrs (GA) before STS (1 µM) or TRAIL (50 ng/mL) addition. Duration of drug treatments varied between 4–24 hours.

### Detection of the Cell Death-associated Functional changes by Flow Cytometry

Data collection was carried out using FACScan flow cytometer (Becton Dickinson) see hereinafter, and analysis of the results was performed by WINLIST software (VERITY Software House, Topsham, USA). The following standard light filters were applied to fluorescence channels: FL1∶530/30 nm; FL2∶585/42 nm; FL3∶650LP. Analysis was performed on the amplitude (height, H) of the fluorescence signal in log scale. A doublet discrimination panel was set on FL2 channel for the detection of FL2A (area) and FL2W (width) of the fluorescence signals are shown in linear scale.

### Assay of PI Uptake of Native Cells Representing the Damage of Plasma Membrane

An increase of permeability of the cell membrane is a characteristic signal for the early phase of necrosis and can be detected using flow cytometry. Treated or non-treated cells were stained directly by adding 0.5 mL PBS containing 5 mM glucose and 10 µg/mL PI to each well, and incubated for a further 15 minutes at 37°C in the CO_2_ incubator. Necrotic (PI positive) cells usually lost light scatter intensity therefore they mixed up with debris in the [FSC, SSC] two-dimensional parameter diagram. For gating necrotic cells out of debris we applied [FSC, FL3H] diagram where necrotic cells contained high amount of DNA could be separated from debris contained low amount of DNA. Gated cells were analyzed on FL2H log scale histograms where the population with low fluorescence intensity represents the PI negative population, while the population displaying high fluorescence represents the membrane ruptured PI positive cells. Data were presented as percentage of cells. In certain experiments, samples were co-labeled with DiOC_6_(3) or Annexin V-FITC together with PI labeling and used for analysis PI uptake.

### Characterization of Phosphatidylserine Distribution in the Plasma Membrane by Flow Cytometry Analysis of Annexin V-FITC and PI Double-labeled Cells

The assay was performed according to the suggestions of the vendor (Alexis Biochemicals, Lausen, Switzerland). Briefly, cells in suspension cultures were stained with an equal volume of PBS containing 5 mM glucose and 10 µg/mL PI in the wells and incubated further for 10 minutes at 37°C. After centrifugation (300×*g*/2 min), cells were suspended in 400 µL binding buffer (10 mM HEPES, 140 mM NaCl, 2.5 mM CaCl_2_) and stained with 5 µL AnnexinV-FITC for 10 minutes in dark. Thereafter an additional 400 µL of binding buffer, containing 1 µg/mL PI was add to the samples and measured immediately. The [FSC, FL3H] diagram was applied for gating and the gated cells were analyzed in [FL1H, FL2H] log scale two dimensional diagram. Auto-fluorescence of the cells was positioned in first decade, and compensation for FITC fluorescence in the FL2 channel was applied. Data were presented as percentage of cells in the marked regions of the diagram.

### Changes of Mitochondrial Transmembrane Potential were Characterized by the DiOC_6_(3) Uptake Method

Samples were prepared for the measurements according to Darzynkiewicz and Bender [61], with minor modifications. Pre-treated cells were stained with an equal volume of PBS containing 5 mM glucose, 10 nM DiOC_6_(3) and 10 µg/mL PI directly in the wells and incubated further for 15 minutes at 37°C and analyzed without further washing steps by FACS. Fluorescent signals of the cells were gated in [FSC, FL3H] diagram, analyzed on FL1H log scale histograms and the data were presented as fluorescence intensity of the whole gated cell population. High ΔΨ, but PI negative cells were considered as functioning mitochondria–containing, surviving population of cells.

### Functioning Lysosomal Compartments are Characterized by the Red Fluorescence of Acridine Orange in the Acidic Environment of Lysosomes

Treated cells were stained with an equal volume of PBS containing 5 mM glucose and 5 µg/mL AO directly in the wells and incubated for a further 15 minutes at 37°C. The pelleted cells (300×*g*/2 minutes) were resuspended in 1 mL of 5 mM glucose containing PBS and analyzed by FACS immediately. Cells were gated in [FL1H, FL3H] diagram for discriminating cells with high DNA content from debris with low amount of DNA. The gated populations were analyzed on FL3H log scale histograms and the data were presented as fluorescence intensity of the whole population.

### Determination of Oligonucleosomal DNA Fragmentation by the Measurement of Sub-G1 Population of Cells

Apoptosis was characterized by measuring the sub-G1 pool of cells as an indication of DNA fragmentation. Samples were prepared according to Gong *et al.* [62]. Briefly, treated cells were pelleted (300×*g*/2 min) and the cells were suspended in 1 mL 70% ethanol (−20°C), and were fixed at room temperature for a half hour. The ethanol-fixed cells were sedimented again and their broken, oligonucleosomal sized DNA content were extracted by treating the cells with 750 µL of extraction buffer containing 200 mM Na_2_HPO_4_/citric acid (pH = 7.8) buffer containing 10 µg/mL RNAse A, for 15 minutes. The DNA remaining inside the permeabilized cells were stained with 5 µg/mL of PI for at least 10 minutes before FACS analysis. Cells were gated in (FSC, FL2H) diagram for discriminating debris and analyzed on FL2H log scale histogram as percentage of cells in the marked, sub-G1 region.

### Agarose Gel Electrophoresis

DNA fragmentation was analyzed as described earlier [63]. Briefly, U937 cells (one million cells per sample) were treated for 20 hrs as indicated at the legends of Figure S2H and Figure S4H. Then cells were collected, washed with PBS and resuspended in 300 µl of a solution, containing 10 mM Tris-HCl, pH 7.5, 1 mM EDTA, 0.15 M NaCl, 1% SDS supplemented with 0.2 mg/ml of proteinase K and incubated overnight at 37^o^C. Samples were phenol-chloroform extracted once and their DNA content were precipitated in ethanol, pelleted and redissolved in 50 µl of TE buffer containing 0.2 mg/mL DNAse free RNAse and incubated for an hour at 37^o^C. DNA was electrophoresed in 1.5% agarose gel in TBA buffer, stained with ethidium bromide and DNA was detected under UV light.

### Light Microscopic Studies

Cytospin preparations (10^5^ cells) were fixed in methanol, stained with hematoxylin-eosin, were dehydrated with subsequent extractions with ethanol, acetone and xilol. The morphological changes of apoptosis were studied by light microscopy at 400x magnification. Cells were categorized according to the recommendations of Kroemer *et al.* [64] as follows: stage I apoptotic cells have moderately condensed nuclei (including smooth nuclei too); stage II apoptotic cells have full blown nuclear condensation (pyknosis) and formation of nuclear apoptotic bodies (karyorrhexis). Necrosis was characterized by cell swelling and blurred plasma membrane. Ghost cells with washed out cytoplasmic contents were identified as being in the late necrotic stage.

### Fluorescent Microscopic Studies

Treated U937 cells were pelleted (300×*g/*3 min) and were stained with 2 µM Hoechst dye (332581) in 1 mL 5 mM glucose containing PBS to distinguish between apoptotic and necrotic cells. After an incubation for 30 minutes at 37°C, cells were pelleted (300×*g/*3 minutes) again and resuspended in 10 µL of 5 mM glucose and 10 µM PI containing PBS and were immediately photographed under a fluorescence microscope (Nikon Eclipse E 400, Japan) at 400x magnification using a SPOT Jr Camera. Excitation wavelength of 330–380 nm was applied for Hoechst dye and 450–490 nm for PI. Apoptotic cells were identified on the basis of morphologic changes in their nuclear assembly by observing chromatin condensation and fragment staining by the Hoechst dye. Secondary necrotic cell were identified based on positive staining with PI and apoptotic nuclear morphology with Hoechst dye. In each case at least four microscopic fields were photographed randomly. The experiments were repeated at least twice.

### Western Blot Representation of PARP-1 and RIPK1 Cleavage

Western blot analysis of the proteolytic degradation of PARP-1 and RIPK1 was carried as published [65]. U937 cells were treated as indicated at the legends of Figure 1B, Figure 3A, and Figure S4I. After treatment cells were collected, washed with PBS and resuspended in 50 µl of Laemmli sample buffer. After boiling the samples their 20 µl aliquots were electrophoresed in a 10% SDS-PAGE gel and blotted onto nitrocellulose. After blocking membranes with 3% milk in PBS overnight, specific antibodies against PARP-1 (#9542, Cell Signaling Technology) or RIPK-1 (BD 610458) were added, incubated for 3 hours and membranes were washed exhaustively with 1% milk containing PBS (25 ml of each washing step) and with PBS twice. Proteins were visualized by ECL. After immuno-detection of RIPK-1 protein levels in cells, blots were stripped and reprobed with an anti GAPDH antibody (Santa Cruz, (D-6) sc-166545). The intensities of GAPDH bands served as loading controls.

### DEVD-ase Activity Assay

Aliquots of cells (5×10^5^ cells/mL) incubated for different durations of time with drugs were withdrawn centrifuged and washed with PBS twice (300×*g*/2 minutes) and finally were suspended in 100 µl of caspase buffer (50 mM HEPES (pH = 7.4), 100 mM NaCl, 0.1% (w/v) CHAPS, 10% (w/v) sucrose and 10 mM DTT. After transferring samples into wells of 96-well plates, Triton X-100 (final concentration of 0.2%) were added, and cell lysis was completed by repeated up and down pipetting of the cells. Caspase substrate z-DEVD-AMC (20 µM) was admixed, and fluorescence intensities of the liberated AMC were recorded for 15 minutes in Fluoroskan Ascent fluorescence plate reader (Thermo Fisher Scientific, Waltman MA, USA). Excitation wavelength was 380 nm and emission is measured at 445 nm. Protease activity was expressed as the slope of the AMC fluorescence curves.

### Statistics

Statistical analysis and significance of differences in comparable values were calculated by applying Student’s t-probe (two tailed, two sample unequal variance) and the indicated significance of differences in the graphs are: P<0.05 (*); P<0.01 (**); P<0.001 (***).

## Supporting Information

Figure S1 **TRAIL induces necrotic type DNA degradation in the presence of caspase inhibitor.** Representative histograms of PI stained, ethanol-fixed U937 cells, detected by flow cytometry (sub-G1 technique). Inserted values indicate the percentage of cells in the marked regions. U937 cells were exposed to TRAIL (50 ng/mL) in the presence or absence of zVD (5 µM) and Nec (10 µM) or GA (1 µM) for 20 hrs (n = 3).
(TIF)

Figure S2 **STS induces necrotic type DNA degradation in the presence of caspase inhibitor.** (A) Nec reduced the STS-induced necroptosis in a concentration-dependent manner after 8 hrs incubation time – representative experiment. Cells were exposed to STS (1 µM) and varying concentrations of Nec (0–50 µM) in the presence of zVD (5 µM) for 8 hrs. Percentage of PI positive cells was determined. (B-F) STS induced DNA fragmentation. U937 cells were treated with STS (1 µM) in the presence of zVD (5 µM) for 12 hrs or 20 hrs. Cells were pre-treated with Nec (10 µM, 1 hr) or GA (1 µM, 4 hrs). (B, D) Representative fluorescent microscopic images (400x) of Hoechst/PI double stained U937 cells (n = 2). Scale bar on the first subfigure applies to all the figures in the panel. (C, E, F) Representative histograms of PI stained, ethanol-fixed cells detected by flow cytometry (sub-G1 technique) (n = 3 for 8 hrs with Nec, n = 13 for 20 hrs with Nec and n = 4 for 20 hrs with GA treatments). Inserted values indicate the percentage of cells in the marked regions. (G) STS-induced caspase (DEVDase) activity in U937 cells. The ordinate shows the slope of the measured DEVDase activity curves of a representative experiment carried out in triplicates. (H) STS-induced DNA fragmentation. Agarose gel electrophoresis was performed to detect the DNA ladder formation. Cells were treated as indicated for 20 hrs (representative of n = 2).
(TIF)

Figure S3 **TRAIL and STS induce MLKL-independent DNA fragmentation and secondary necrosis.** (A) Representative histograms of PI stained, ethanol-fixed U937 cells detected by flow cytometry (sub-G1 technique). Inserted values indicate the percentage of cells in the marked regions. U937 cell were exposed to TRAIL (50 ng/mL) or STS (1 µM) and NSA (0.5 µM) for 20 hrs (n = 3). (B) Morphological signs of apoptosis and necrosis are shown in representative fluorescent microscopic images (400x) of Hoechst/PI double stained U937 cells (representative of n = 2). Scale bar on the first subfigure applies to all the figures in the panel.
(TIF)

Figure S4 **CA inhibits both the TRAIL and STS-induced necroptosis in the presence of caspase inhibitor.** U937 cells were treated either with STS (1 µM) or with TRAIL (50 ng/mL) in the presence or absence of zVD (5 µM) for 20 hrs. Nec (10 µM) or CA (10 µM or as indicated) were added 1 hr before cell death was induced. (A-B) CA reduced the ratio of PI positive cells for STS+zVD or TRAIL+zVD treatment for 20 hrs in a concentration-dependent manner, representative experiments. (C) Column diagram of percentage of cells with high AO fluorescence intensity (n = 2). (D) Column diagram of percentage of cells with high DiOC_6_(3) fuorescence intensity (n = 4). (E-F) Representative histograms of PI stained, ethanol-fixed U937cells detected by flow cytometry (sub-G1 technique). The numbers indicate the percentage of cells in the marked regions (n = 3 for STS and n = 4 for TRAIL). Values are mean±SD. *, P<0.05, **, P<0.01 and ***, P<0.001 calculated by Student’s t-probe. (G-H) STS-induced DNA fragmentation and condensation. (G) Hoechst/PI double staining (400x) and (H) agarose gel electrophoresis was performed with samples treated as indicated for 20 hrs (representative of n = 2). Scale bar on the first subfigure applies to all the figures in the panel. (I) zVD treatment prevents RIPK1 fragmentation triggered by STS. Western blot analysis was performed for the detection of RIPK1 protein level and presence of cleaved fragment due to caspase activity (representative of n = 2).
(TIF)

Figure S5 **PJ-34 does not arrest the TRAIL-induced necroptosis in the presence of caspase inhibitor.** U937 cells were treated with TRAIL (50 ng/mL) in the presence or absence of zVD (5 µM) for 20 hrs. PJ-34 (1 µM) was added 1 hr before cell death induction. (A) Column diagram of percentage of cells with high DiOC_6_(3) fluorescence intensity (n = 4). (B) Column diagram of percentage of cells with high AO red fluorescence intensity (n = 2). (C) Representative histograms of PI stained, ethanol-fixed cells detected by flow cytometry (sub-G1 technique). The numbers indicate the percentage of cells in the marked regions (n = 8).
(TIF)

Text S1 **GA is more potent inhibitor than Nec during STS-provoked necroptosis.**
(PDF)

Text S2 **Autophagy may play role in necroptosis.**
(PDF)

Text S3 **Nec can inhibit necrosis in a RIPK1-independent manner.**
(PDF)

Text S4 **PARP-2 might be involved in the necroptotic signaling pathway.**
(PDF)
