# Supplementary figures and images for: Staurosporine Induces Necroptotic Cell Death under Caspase-Compromised Conditions in U937 Cells
Source: PLoS One. 2012 Jul 31;7(7):e41945. doi: 10.1371/journal.pone.0041945 (PMC3409216; doi:10.1371/journal.pone.0041945)

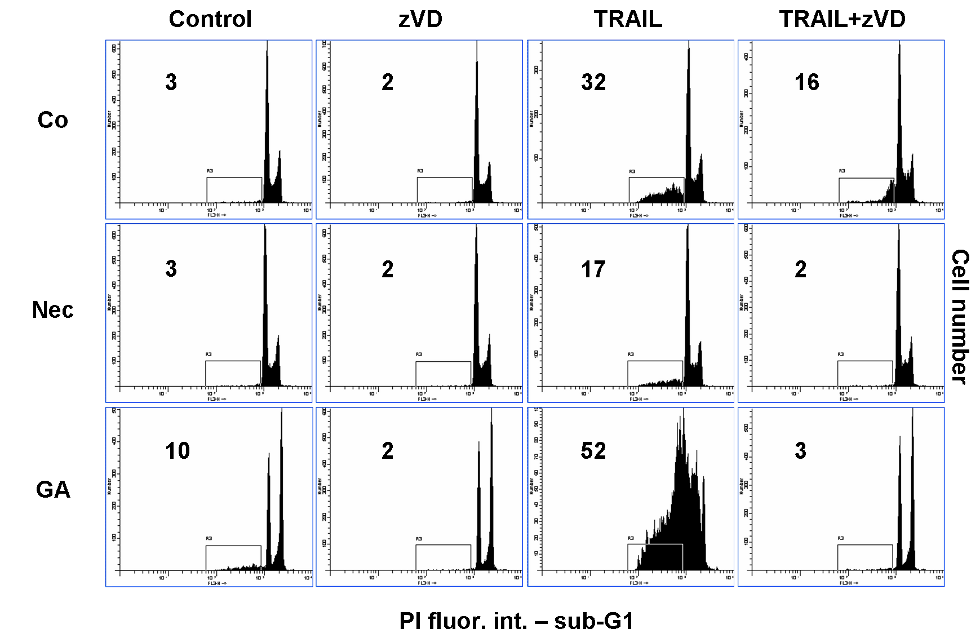

Supplement: Figure S1 — TRAIL induces necrotic type DNA degradation in the presence of caspase inhibitor. Representative histograms of PI stained, ethanol-fixed U937 cells, detected by flow cytometry (sub-G1 technique). Inserted values indicate the percentage of cells in the marked regions. U937 cells were exposed to TRAIL (50 ng/mL) in the presence or absence of zVD (5 µM) and Nec (10 µM) or GA (1 µM) for 20 hrs (n = 3). (TIF) [file pone.0041945.s001.tif]

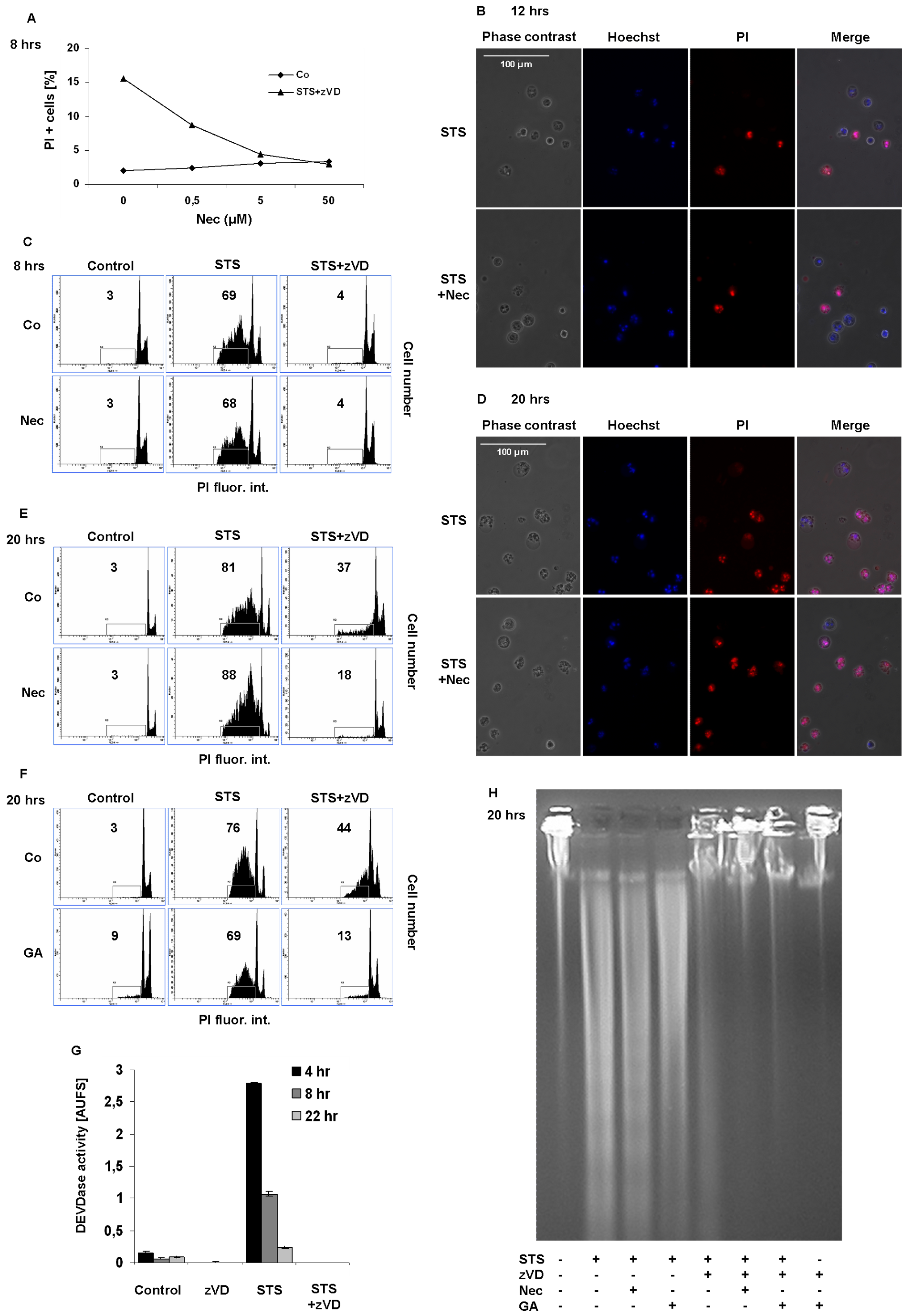

Supplement: Figure S2 — STS induces necrotic type DNA degradation in the presence of caspase inhibitor. (A) Nec reduced the STS-induced necroptosis in a concentration-dependent manner after 8 hrs incubation time – representative experiment. Cells were exposed to STS (1 µM) and varying concentrations of Nec (0–50 µM) in the presence of zVD (5 µM) for 8 hrs. Percentage of PI positive cells was determined. (B-F) STS induced DNA fragmentation. U937 cells were treated with STS (1 µM) in the presence of zVD (5 µM) for 12 hrs or 20 hrs. Cells were pre-treated with Nec (10 µM, 1 hr) or GA (1 µM, 4 hrs). (B, D) Representative fluorescent microscopic images (400x) of Hoechst/PI double stained U937 cells (n = 2). Scale bar on the first subfigure applies to all the figures in the panel. (C, E, F) Representative histograms of PI stained, ethanol-fixed cells detected by flow cytometry (sub-G1 technique) (n = 3 for 8 hrs with Nec, n = 13 for 20 hrs with Nec and n = 4 for 20 hrs with GA treatments). Inserted values indicate the percentage of cells in the marked regions. (G) STS-induced caspase (DEVDase) activity in U937 cells. The ordinate shows the slope of the measured DEVDase activity curves of a representative experiment carried out in triplicates. (H) STS-induced DNA fragmentation. Agarose gel electrophoresis was performed to detect the DNA ladder formation. Cells were treated as indicated for 20 hrs (representative of n = 2). (TIF) [file pone.0041945.s002.tif]

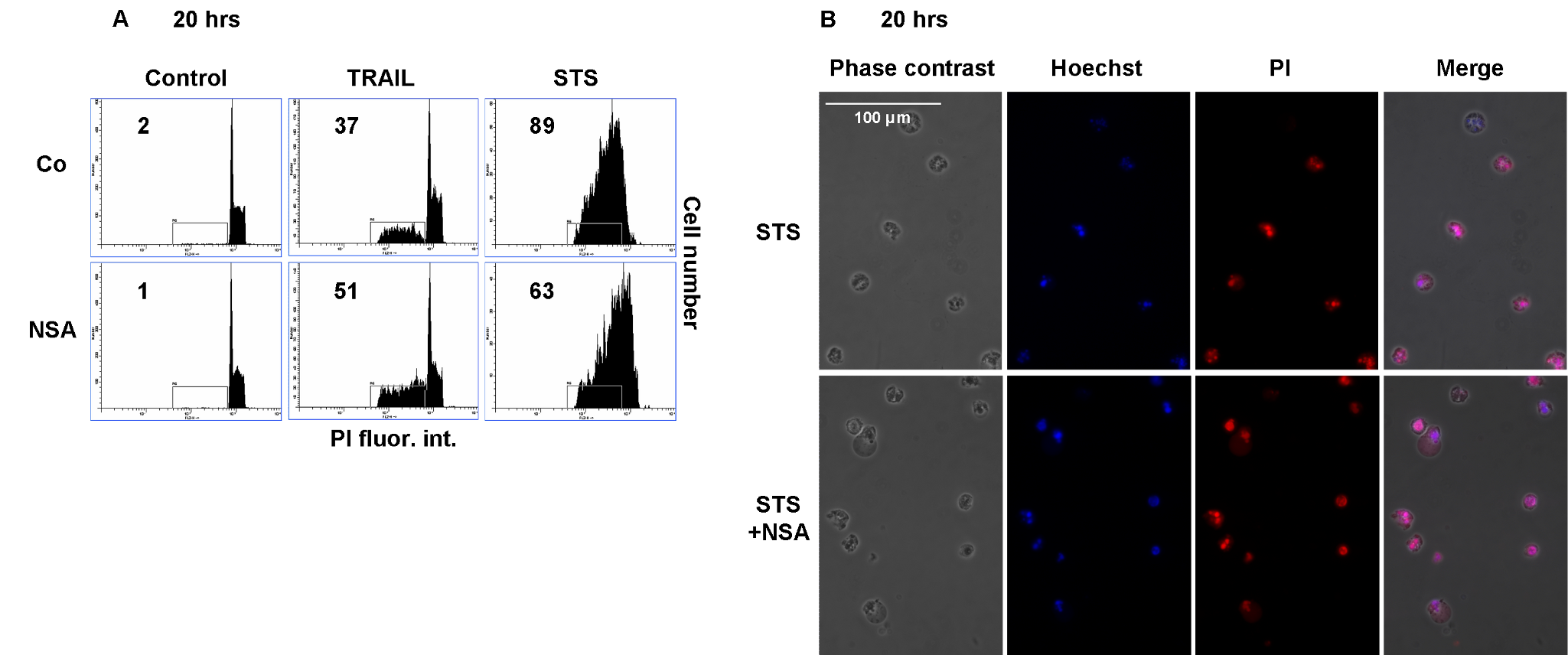

Supplement: Figure S3 — TRAIL and STS induce MLKL-independent DNA fragmentation and secondary necrosis. (A) Representative histograms of PI stained, ethanol-fixed U937 cells detected by flow cytometry (sub-G1 technique). Inserted values indicate the percentage of cells in the marked regions. U937 cell were exposed to TRAIL (50 ng/mL) or STS (1 µM) and NSA (0.5 µM) for 20 hrs (n = 3). (B) Morphological signs of apoptosis and necrosis are shown in representative fluorescent microscopic images (400x) of Hoechst/PI double stained U937 cells (representative of n = 2). Scale bar on the first subfigure applies to all the figures in the panel. (TIF) [file pone.0041945.s003.tif]

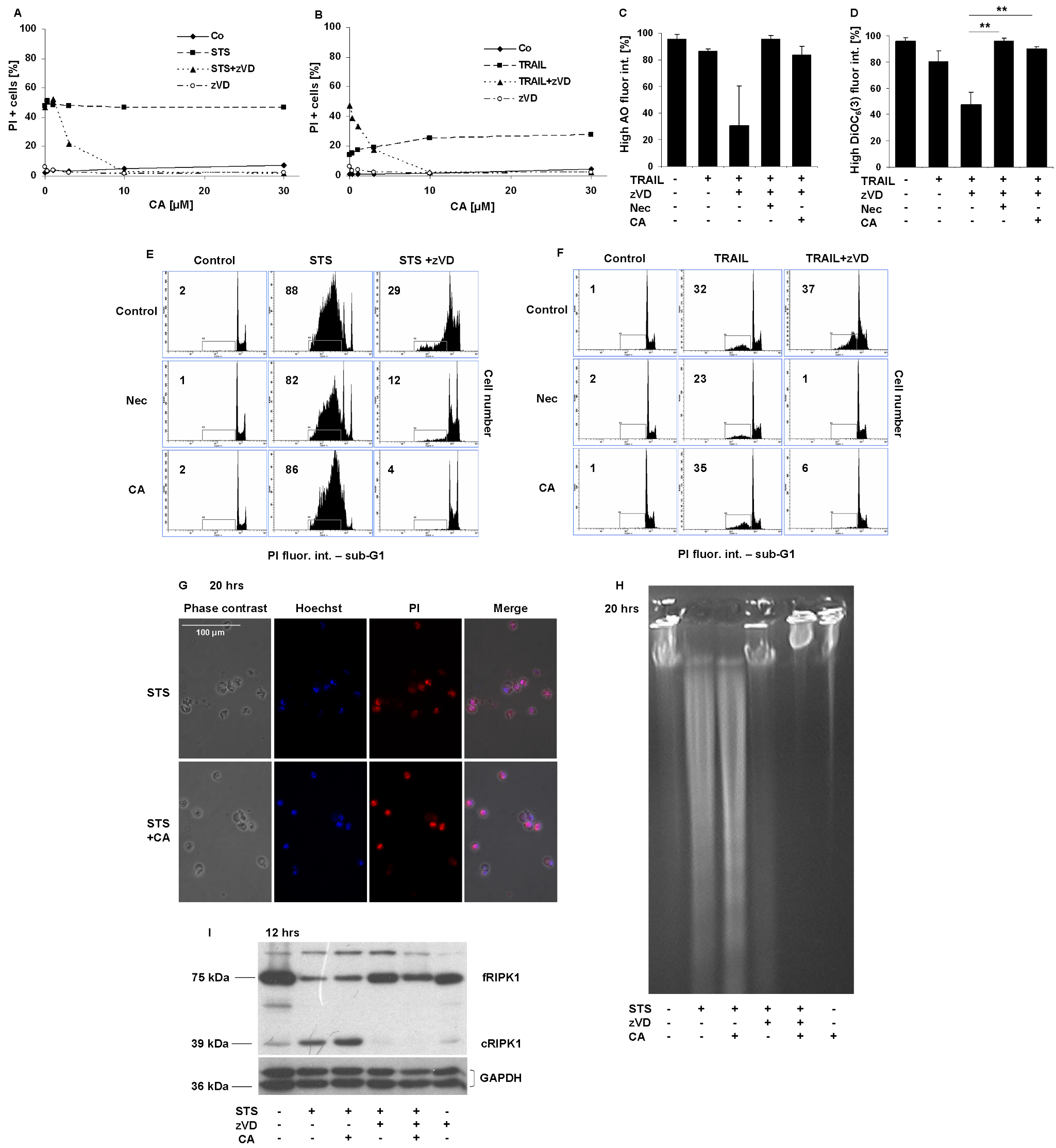

Supplement: Figure S4 — CA inhibits both the TRAIL and STS-induced necroptosis in the presence of caspase inhibitor. U937 cells were treated either with STS (1 µM) or with TRAIL (50 ng/mL) in the presence or absence of zVD (5 µM) for 20 hrs. Nec (10 µM) or CA (10 µM or as indicated) were added 1 hr before cell death was induced. (A-B) CA reduced the ratio of PI positive cells for STS+zVD or TRAIL+zVD treatment for 20 hrs in a concentration-dependent manner, representative experiments. (C) Column diagram of percentage of cells with high AO fluorescence intensity (n = 2). (D) Column diagram of percentage of cells with high DiOC6(3) fuorescence intensity (n = 4). (E-F) Representative histograms of PI stained, ethanol-fixed U937cells detected by flow cytometry (sub-G1 technique). The numbers indicate the percentage of cells in the marked regions (n = 3 for STS and n = 4 for TRAIL). Values are mean±SD. *, P<0.05, **, P<0.01 and ***, P<0.001 calculated by Student’s t-probe. (G-H) STS-induced DNA fragmentation and condensation. (G) Hoechst/PI double staining (400x) and (H) agarose gel electrophoresis was performed with samples treated as indicated for 20 hrs (representative of n = 2). Scale bar on the first subfigure applies to all the figures in the panel. (I) zVD treatment prevents RIPK1 fragmentation triggered by STS. Western blot analysis was performed for the detection of RIPK1 protein level and presence of cleaved fragment due to caspase activity (representative of n = 2). (TIF) [file pone.0041945.s004.tif]

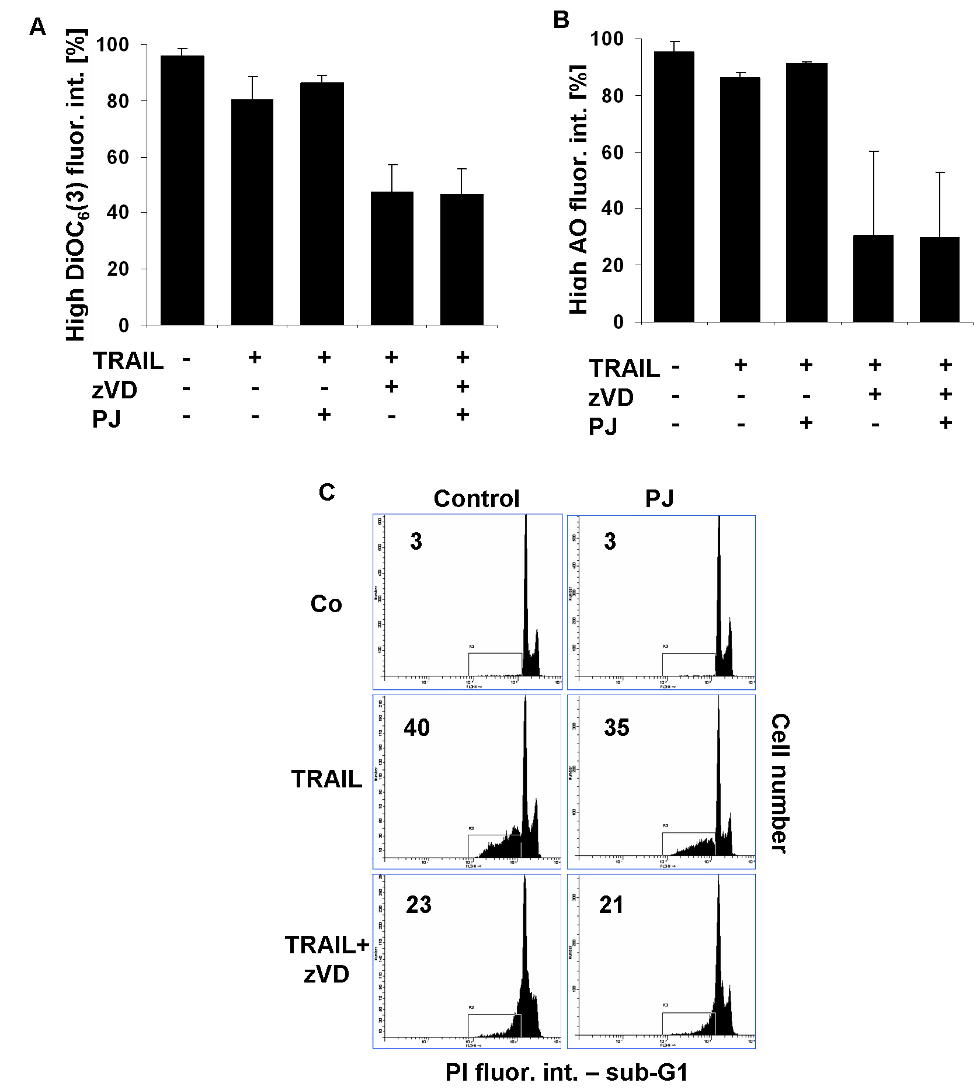

Supplement: Figure S5 — PJ-34 does not arrest the TRAIL-induced necroptosis in the presence of caspase inhibitor. U937 cells were treated with TRAIL (50 ng/mL) in the presence or absence of zVD (5 µM) for 20 hrs. PJ-34 (1 µM) was added 1 hr before cell death induction. (A) Column diagram of percentage of cells with high DiOC6(3) fluorescence intensity (n = 4). (B) Column diagram of percentage of cells with high AO red fluorescence intensity (n = 2). (C) Representative histograms of PI stained, ethanol-fixed cells detected by flow cytometry (sub-G1 technique). The numbers indicate the percentage of cells in the marked regions (n = 8). (TIF) [file pone.0041945.s005.tif]
