# Supplementary material for: Staurosporine Induces Necroptotic Cell Death under Caspase-Compromised Conditions in U937 Cells
Source: PLoS One. 2012 Jul 31;7(7):e41945. doi: 10.1371/journal.pone.0041945 (PMC3409216; doi:10.1371/journal.pone.0041945)
Supplement: Text S1 — GA is more potent inhibitor than Nec during STS-provoked necroptosis. (PDF) [file pone.0041945.s006.pdf]

Supporting information
Text

Text S1

The inhibitory effect of GA was more potent than that of Nec during STS-evoked necroptosis; however, the difference was not significant. This might be the consequence of the inhibition of HSP90 and degradation or loss of function of various other proteins than RIPK1. E.g. the autophagyc protein Beclin-1 was shown to form complex with HSP90 [1]. Parallel processes of necroptosis and autophagy might be inhibited by GA while Nec arrests only the former one.

Reference

1. Xu C, Liu J, Hsu L, Luo Y, Xiang R, et al. (2011) Functional interaction of Hsp90 and Beclin 1 modulates Toll-like receptor-mediated autophagy. FASEB Journal 25: 2700-2710.
