# Supplementary material for: Staurosporine Induces Necroptotic Cell Death under Caspase-Compromised Conditions in U937 Cells
Source: PLoS One. 2012 Jul 31;7(7):e41945. doi: 10.1371/journal.pone.0041945 (PMC3409216; doi:10.1371/journal.pone.0041945)
Supplement: Text S2 — Autophagy may play role in necroptosis. (PDF) [file pone.0041945.s007.pdf]

Supporting information
Text

Text S2

Degterev *et al.* have shown that autophagy is a common downstream consequence of necroptosis and acts as a repair and energy production process, but inhibition of autophagy has no effect on necroptosis [1]. On the contrary, Bonapace *et al.* reported that inhibition of autophagy blocked the necroptotic process [2]. Autophagy may play role parallel or sequential to necroptosis after treatments with STS and zVD, as 3-methyladenine partially (like Nec) inhibited the PI staining in U937 cells (Dunai *et al.*, unpublished result).

References

1. Degterev A, Huang Z, Boyce M, Li Y, Jagtap P, et al. (2005) Chemical inhibitor of nonapoptotic cell death with therapeutic potential for ischemic brain injury. Nature chemical biology 1: 112-119.
2. Bonapace L, Bornhauser BC, Schmitz M, Cario G, Ziegler U, et al. (2010) Induction of autophagy-dependent necroptosis is required for childhood acute lymphoblastic leukemia cells to overcome glucocorticoid resistance. Journal of Clinical Investigation 120: 1310-1323.
