# Supplementary material for: Staurosporine Induces Necroptotic Cell Death under Caspase-Compromised Conditions in U937 Cells
Source: PLoS One. 2012 Jul 31;7(7):e41945. doi: 10.1371/journal.pone.0041945 (PMC3409216; doi:10.1371/journal.pone.0041945)
Supplement: Text S3 — Nec can inhibit necrosis in a RIPK1-independent manner. (PDF) [file pone.0041945.s008.pdf]

Supporting information
Text

Text S3

In a recent publication STS was shown to induce cell death in cultured rat cortical astrocytes, that can be arrested by 100 µM Nec [1]. However in a former publication Cho *at al.* demonstrated that Nec can inhibit necrosis in a RIPK1-independent manner in L929 cell line at around 20-50 µM Nec concentration when cell death was induced by TNFα administration. Thus, care should be taken when results obtained with this inhibitor are interpreted [2]. To reflect on this, throughout our experiments we used lower concentration of Nec (namely 10 µM).

References

1. Simenc J, Lipnik-Stangelj M (2012) Staurosporine induces different cell edath form in cultures rat astrocytes. Radiology and Oncology doi:10.2478/v10019-012-0036-9.
2. Cho Y, McQuade T, Zhang H, Zhang J, Chan FKM (2011) RIP1-dependent and independent effects of necrostatin-1 in necrosis and T cell activation. PLoS ONE 6: e23209.
