# Supplementary material for: Staurosporine Induces Necroptotic Cell Death under Caspase-Compromised Conditions in U937 Cells
Source: PLoS One. 2012 Jul 31;7(7):e41945. doi: 10.1371/journal.pone.0041945 (PMC3409216; doi:10.1371/journal.pone.0041945)
Supplement: Text S4 — PARP-2 might be involved in the necroptotic signaling pathway. (PDF) [file pone.0041945.s009.pdf]

Supporting information
Text

Text S4

It is also worth mentioning that PJ-34 is active site inhibitor of PARP-1 and PARP-2 [1]. Using siRNA gene ablation screen it was shown that PARP-2 is involved in the necroptotic process [2]. It is possible that not the enzymatic activity of PARP-2 but an adapter function is involved in the cell death process, only its presence as a protein is needed as a building block of a signaling protein complex.

References

1. Soriano FG, Virag L, Szabo C (2001) Diabetic endothelial dysfunction: role of reactive oxygen and nitrogen species production and poly(ADP-ribose) polymerase activation. Journal of Molecular Medicine 79: 437-448.
2. Hitomi J, Christofferson DE, Ng A, Yao J, Degterev A, et al. (2008) Identification of a Molecular Signaling Network that Regulates a Cellular Necrotic Cell Death Pathway. Cell 135: 1311-1323.
